# The Intensive Family & Adolescent Eating Disorders (IFAED) day program: a collaboration between Sydney Children's Hospital Network Eating Disorder Service and The Butterfly Foundation

**DOI:** 10.1186/2050-2974-3-S1-O8

**Published:** 2015-11-23

**Authors:** Julian Baudinet, Andrew Wallis, Lisa Dawson, Elaine Tay, Dale Greenwood, Caitlin McMaster, Andrew Kennedy, Sloane Madden, Jane Miskovic-Wheatley

**Affiliations:** 1 The Children's Hospital at Westmead, Westmead, NSW, Australia

## 

The Sydney Children's Hospital Network Eating Disorder Service (EDS) at The Children's Hospital at Westmead (CHW) is a tertiary service that offers a range of family focused treatment options for young people with an eating disorder. The Intensive Family and Adolescent Eating Disorder (IFAED) Day Program is a new program that has been operating since September 2014, which targets young people and their families who have not been responding to standard treatment or who present with additional complexity.

The IFAED program is designed as a treatment option that is more intensive than outpatient treatment, but less restrictive than overnight inpatient care. It operates five days per week and offers integrative and multidisciplinary care combining medical review, psychological treatment, meal support, secondary education and family support, including multiple family therapy.

Theoretically, the program aims to strengthen and support families by increasing treatment intensity and solidarity to address barriers that are preventing progress. Interventions are provided for both families and young people. Underpinning the program is the belief that families are essential to recovery with weekly family therapy and parent groups enabling the family to be more responsive to the young person's behavioural and emotional needs. Group therapy for the young people targets skills to manage distress, improve flexible thinking, explore adolescent issues and improve communication skills. These interventions aim to improve coping and help the family support to be more effective. This presentation will outline the current IFAED Day Program structure and content, theoretical underpinnings and some preliminary outcome data.

